# Stimulation of soluble guanylyl cyclase protects against obesity by recruiting brown adipose tissue

**DOI:** 10.1186/2050-6511-16-S1-A6

**Published:** 2015-09-02

**Authors:** Alexander Pfeifer, Jennifer Etzrodt, Linda S Hoffmann

**Affiliations:** 1 Institute of Pharmacology and Toxicology, University Hospital Bonn, University of Bonn, Bonn, Germany

## Clinical background

Obesity has reached pandemic dimensions and novel pharmacological therapies are urgently needed. Obesity is characterized by excessive fat storage in white adipose tissue (WAT), because of a positive energy balance. In contrast to WAT, brown adipose tissue (BAT) dissipates energy and produces heat – a process known as non-shivering thermogenesis. To identify novel BAT-centered antiobesity therapies, we studied the role of soluble guanylyl cyclase (sGC) in BAT. sGC produces the second messenger cyclic GMP (cGMP) after stimulation with nitric oxide.

Here, we used a small molecule that stimulates sGC in a heme–dependent manner. Treatment of mice with the sGC stimulator during a high fat diet protected against weight gain and improved metabolic changes. Notably, stimulation of sGC induced weight loss also in already established obesity. Mechanistically, the sGC stimulator enhanced expression of thermogenic genes and induced “browning” (i.e. the expression of brown adipocyte-specific markers) of murine and human adipocytes. sGC stimulation increased lipid uptake into BAT, and caused an increase in whole body energy expenditure.

## Conclusion

Taken together, sGC is a potential pharmacological target for the treatment of obesity and its comorbidities.

